# Genomic Surveillance Reveals Clusters of *Plasmodium falciparum* Antimalarial Resistance Markers in Eswatini, A Low-Transmission Setting

**DOI:** 10.4269/ajtmh.25-0487

**Published:** 2026-05-19

**Authors:** Nomcebo Nhlengethwa, Andrés Aranda-Díaz, Sibonakaliso Vilakati, Alfred Hubbard, Isobel Routledge, Sonja B. Lauterbach, Takalani I. Makhanthisa, Faith De Amaral, Mukosha Chisenga, Brighton Mangena, Chadwick Sikaala, John Chimumbwa, Quinton Dlamini, Jaishree Raman, Jennifer L. Smith, Sabelo V. Dlamini

**Affiliations:** ^1^ Laboratory Department, National Malaria Programme, Manzini, Eswatini;; ^2^ EPPIcenter Research Program, Department of Medicine, University of California, San Francisco, California, USA;; ^3^ Malaria Elimination Initiative, Institute of Global Health Sciences, University of California, San Francisco, California, USA;; ^4^ ISGlobal, Barcelona, Spain;; ^5^ Department of Epidemiology, Johns Hopkins University Bloomberg School of Public Health, Baltimore, Maryland, USA;; ^6^ Laboratory for Antimalarial Resistance Monitoring and Malaria Operational Research, Centre of Emerging Zoonotic and Parasitic Diseases, National Institute for Communicable Diseases, Johannesburg, South Africa;; ^7^ SADC Malaria Elimination Eight Secretariat, Windhoek, Namibia;; ^8^ Wits Research Institute for Malaria, University of Witwatersrand, Johannesburg, South Africa;; ^9^ University of Pretoria Institute for Sustainable Malaria Control, University of Pretoria, Pretoria, South Africa;; ^10^ Department of Epidemiology & Biostatistics, University of California, San Francisco, California;; ^11^ Parasitology Department, Faculty of Health Sciences, University of Eswatini, Mbabane, Eswatini

## Abstract

The elimination of *Plasmodium falciparum* malaria in Eswatini remains elusive because of ongoing importation and sustained local transmission. The current status of antimalarial and diagnostic resistance in the country remains unknown. Genomic surveillance can complement routine surveillance by characterizing the prevalence and distribution of resistance markers and revealing granular patterns of transmission. Between March and December 2023, dried blood spots, demographic data, and travel history were collected from individuals who tested positive for malaria on a rapid diagnostic test (RDT) and from symptomatic RDT-negative individuals across Eswatini. Multiplexed amplicon deep sequencing was used to genotype 12 genes associated with antimalarial resistance, detect histidine-rich protein (*hrp*) *2*/*3* deletions, estimate genetic relatedness, and detect non-*falciparum* species. Data from 437 samples revealed significant clustering of parasites carrying resistance markers. A validated marker of artemisinin partial resistance, *kelch13* P553L, was identified in a cluster of four infections (0.9% of all samples), likely linked to importation. The dihydropteroate synthase/dihydrofolate reductase sextuple mutant haplotype, associated with high-level sulfadoxine–pyrimethamine resistance, was found in 7.8% of the samples, also within clusters. The multidrug resistance 1 N86 genotype associated with reduced susceptibility to lumefantrine was nearly fixed. More than 60% of the infections were polyclonal, with higher complexity observed in imported cases. No *hrp2*/*3* deletions were detected; most false-negative RDTs were attributed to subpatent parasitemia. Non-*falciparum* coinfections were rare (<1%). These findings shed light on the dynamics of resistance emergence in low-transmission settings. Integrating routine genomic surveillance into national malaria programs is essential for detecting, tracking, and responding to resistance threats in countries nearing elimination.

## INTRODUCTION

Malaria elimination in Eswatini, a southern African country targeting elimination by 2025, remains elusive, in part because of persistent local transmission and frequent importation of cases, which fuel periodic upsurges.^1^ Although the national malaria prevalence remains low, malaria still affects hundreds of individuals each year.^1^ Between 2021 and 2022, Eswatini reported an average of 470 malaria cases per year, including 79 severe cases and three deaths annually according to unpublished data from the Eswatini National Malaria Programme (NMP) Malaria Surveillance Database System (MSDS). To curb transmission, Eswatini has implemented a combination of interventions that target both the vector and the parasite, including indoor residual spraying (IRS) and the prompt treatment of diagnosed infections with artemisinin-based combination therapies (ACTs). The effectiveness of interventions targeting the parasite depends on timely case detection and effective treatment. However, this effectiveness is increasingly threatened by the emergence and spread of *Plasmodium falciparum* (*P. falciparum*) strains that are resistant to antimalarial drugs and diagnostics across Africa.^2^^,^^3^

Artemisinin partial resistance (ART-R), characterized by delayed parasite clearance after ACT treatment, once confined to the Greater Mekong Subregion, has independently emerged in countries in East Africa and the Horn of Africa.^4^^–^^6^ Although resistance to ACT partner drugs has not been confirmed in Africa, as it has in Southeast Asia, increasing reports of ACT treatment failures and reduced partner drug susceptibility in Africa are concerning.^7^^–^^9^ Additionally, *P. falciparum* strains undetectable by the widely used falciparum-specific histidine-rich protein 2 (HRP2)-based rapid diagnostic tests (RDTs) are now prevalent in the Horn of Africa.^10^^,^^11^ These developments emphasize the need to closely monitor antimalarial drug and diagnostic resistance to ensure that effective elimination strategies are in place.

Antimalarial drug resistance is mediated by mutations in the parasite genome that reduce drug sensitivity or impair the parasite’s clearance rate, potentially leading to treatment failure. Artemisinin partial resistance is associated with single-nucleotide polymorphisms (SNPs) in the *kelch13* gene, particularly amino acid substitutions in the propeller domain of the Kelch13 protein.^12^ Over the last decade, SNPs in the *kelch13* gene with clinical or laboratory evidence of ART-R have been increasingly reported in Ethiopia, Eritrea, Tanzania, Uganda, and Rwanda.^10^^,^^13^^–^^17^ Mutations in other genes, including the chloroquine resistance transporter (*crt*) and multidrug resistance 1 (*mdr1*), are associated with reduced sensitivity to the ACT partner drugs, lumefantrine, piperaquine, and amodiaquine.^18^^,^^19^ Copy number variations in the *plasmepsin 2*/*3* and *mdr1* genes, although less common in Africa, have been associated with piperaquine and mefloquine resistance, respectively.^20^^,^^21^ Resistance to sulfadoxine and pyrimethamine results from the accumulation of mutations in the dihydropteroate synthase (*dhps*) and dihydrofolate reductase (*dhfr*) genes, respectively, which are widely prevalent across Africa and exhibit distinct geographic patterns.^22^^–^^24^ Chloroquine resistance, primarily mediated by the *crt* K76T genotype and modulated by additional *crt* and *mdr1* mutations, has declined since chloroquine was withdrawn as a first-line *falciparum* malaria treatment in Africa.^25^^–^^30^

Accurate diagnosis is critical for effective case management and detecting index cases for reactive interventions. Deletions in the genes encoding *hrp2* and *hrp3* compromise the performance of HRP2-based RDTs and are prevalent in Ethiopia and Eritrea.^10^^,^^11^^,^^31^ False-negative RDT results may also arise from parasite densities below the limit of detection, which is common in low-transmission settings, or from high parasite loads that cause a prozone-like effect.^32^^,^^33^

Malaria molecular surveillance can serve as an early warning system by detecting hotspots of antimalarial drug or diagnostic resistance, guiding the implementation of therapeutic efficacy surveys, and informing treatment policy in areas with declining drug efficacy.^34^ Genomic technologies, including targeted amplicon deep sequencing, provide a cost-efficient method of generating molecular data from samples collected through passive or active surveillance, especially when integrated into routine surveillance platforms.^35^ The only malaria molecular surveillance study in Eswatini, which was conducted in 2010, revealed mutation prevalence consistent with the widespread historical use of chloroquine and sulfadoxine–pyrimethamine (SP).^36^ Since artemether–lumefantrine was adopted as a first-line treatment for uncomplicated malaria in 2010, no follow-up investigations or therapeutic efficacy studies have been conducted.^37^

In addition to monitoring drug and diagnostic resistance, parasite genetics can be used to estimate within-host and population-level genetic diversity, providing insights into transmission intensity and dynamics.^38^^–^^41^ When linked to case data, parasite genetic relatedness can be estimated to reveal granular patterns of local transmission, identify clusters of closely related infections, and help reconstruct transmission chains.^42^^–^^45^

To provide an updated overview of antimalarial drug and diagnostic resistance marker prevalence in Eswatini, malaria parasite genomic surveillance was conducted as part of the Genomics for Malaria in the Elimination 8 (GenE8) initiative, which is a regional effort aimed at strengthening malaria genomic surveillance in five Southern African countries (Angola, Eswatini, Namibia, South Africa, and Zambia). In the present study, the prevalence of antimalarial resistance markers in Eswatini is reported, and clusters of infections carrying resistance markers are characterized using parasite genomics, demographic characteristics, and host risk factors.

## MATERIALS AND METHODS

### Study site.

The present study included all four administrative regions of Eswatini, a landlocked country bordered by Mozambique to the east and South Africa to the north, west, and south (Figure 1A). Eswatini has a population of ∼1.2 million, with ∼30% residing in malaria-receptive areas—defined by the presence of malaria vectors (*Anopheles* mosquitoes) and ecological and climatic factors that encourage malaria transmission—primarily located in the lowveld areas of the eastern Lubombo and Hhohho Regions.^1^^,^^46^^,^^47^ Malaria transmission is seasonal, typically occurring between September and May, with most cases reported between January and May.^1^ The annual parasite index for the 2021–2022 season was ∼0.7 cases/1,000 population according to unpublished data from the NMP MSDS. The predominant malaria parasite species is *P*. *falciparum*, with non-*falciparum* infections rarely detected.^48^^,^^49^ The primary mosquito vector is *Anopheles arabiensis*.^1^

**Figure 1.**
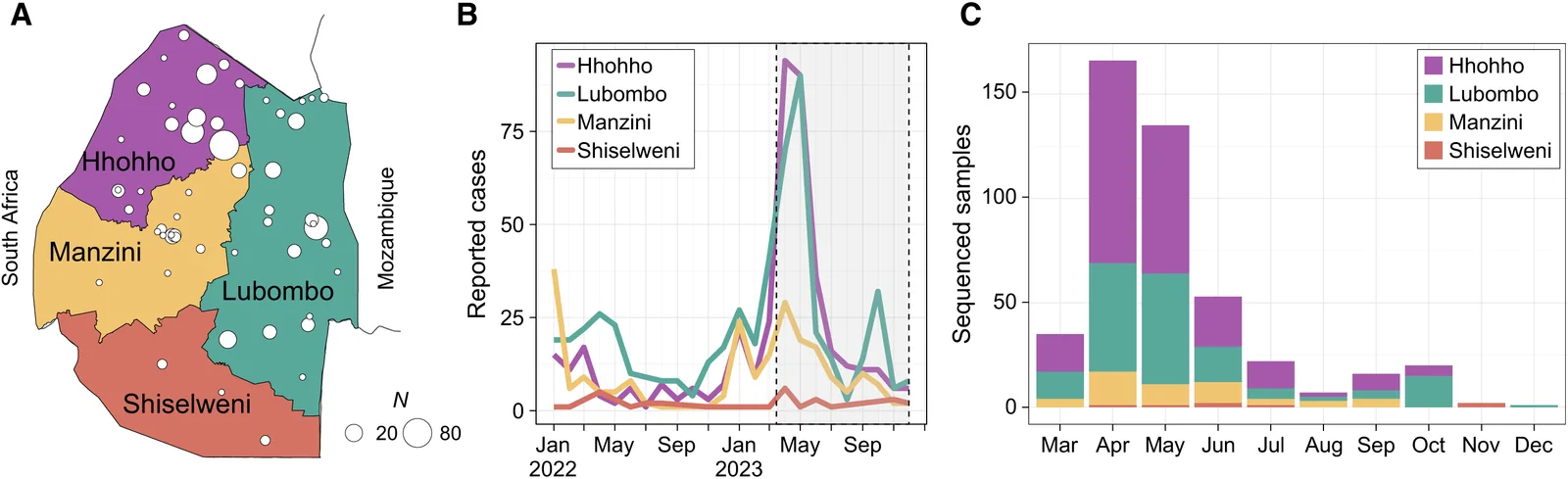
Study site. (**A**) Map of Eswatini and health facilities with sequenced samples. The circle size indicates the number of samples sequenced from each health facility (*N*). (**B**) Total number of reported malaria cases in Eswatini in 2022 and 2023. The study period for this report is highlighted in a dashed box. (**C**) Number of sequenced samples stratified by month.

Of the 365 health facilities in Eswatini’s healthcare system, 265 offer malaria diagnosis and treatment services. Diagnosis is primarily based on an RDT (Parascreen, Zephyr Biomedicals [Goa, India], or Meriscreen, Meril Diagnostics [Gujarat, India]) that detects *P*. *falciparum* HRP2 and pan-*Plasmodium* lactate dehydrogenase (*ldh*) antigens (Pf/Pan). Microscopy is reserved for further analysis at facilities with laboratory capacity.^48^ Uncomplicated malaria cases are treated using artemether–lumefantrine combined with a single low dose of primaquine, whereas severe cases are treated with intravenous or intramuscular artesunate.^50^ Mefloquine- or atovaquone–proguanil-based chemoprophylaxis is provided to travelers to endemic countries at no cost.^50^

Proactive and reactive case detection is conducted in receptive areas. Proactive case detection during outbreaks is targeted to high-risk locations such as farms, border posts, and identified transmission hotspots and is based on available programmatic resources and competing priorities. In 2022, 845 individuals were screened via proactive case detection, yielding one RDT-positive case according to unpublished data from the NMP MSDS. Reactive case detection is routinely conducted in all households located within a 500-meter radius of the household of an index case. Eswatini does not currently implement mass drug administration campaigns. In 2022, 59 index cases led to testing of 1,958 individuals, yielding 28 RDT-positive cases according to unpublished data from the NMP MSDS.

In addition to case management, the country’s malaria elimination strategies include annual targeted IRS (rotating Actellic® and Icon® insecticides [Syngeta Group, Basel, Switzerland]) in receptive areas and reactive spraying around incident cases. Cone bioassays have confirmed the absence of resistance to the insecticides used for vector control in Eswatini.^1^ The distribution of long-lasting insecticidal nets is limited to high-risk groups not protected by IRS (i.e., individuals engaged in agricultural activities in the forests).

### Study design and field procedures.

The present cross-sectional study was conducted as part of the Regional GenE8 project and was integrated into Eswatini’s routine malaria surveillance system. All participants aged over 2 years who tested positive for malaria via Pf/*Pan* RDT or microscopy through passive, reactive, or proactive case detection between March and December 2023 were targeted for inclusion in the study (Figure 1). On the basis of historical data (590 cases in 2019), up to 900 malaria-positive samples were anticipated for sequencing. An additional 200 individuals who tested negative for malaria via RDT but displayed symptoms consistent with malaria were targeted to assess *hrp2*/*3* deletions. Children aged less than 2 years old and individuals who had received treatment or chemoprophylaxis in the preceding 14 days were excluded from the study.

Written informed consent was obtained from all participants before enrollment. As part of routine case investigation activities, enrolled participants were interviewed using an electronic questionnaire to collect demographic data, clinical symptoms, travel history, and occupation. Collected data were uploaded to the NMP MSDS, which serves as a central repository for all malaria-related intervention data. Capillary blood samples were collected via finger-prick onto filter paper strips (Cytiva Whatman 3MM Chr [Cytiva, Marlborough, MA]) and labeled with unique barcodes linking survey and laboratory data. Dried blood spots (DBSs) were air-dried, stored in hard paper cards within plastic bags with desiccant at room temperature, and transported to the NMP laboratory in Manzini within a week of collection. In cases in which DBS collection was not possible, the RDT cassette was labeled and stored under the same conditions. All samples were stored at –20 degrees in the NMP laboratory before being shipped to the regional sequencing hub at the National Institute for Communicable Diseases in South Africa.

### Sample processing and genomic assays.

Parasite densities of genomic DNA extracted from a 6 mm DBS disc or the RDT nitrocellulose strip using the Chelex-Tween 20 method were estimated using the *var* gene acidic terminal sequence (varATS) quantitative real-time polymerase chain reaction (qPCR) assay, calibrated with a standard curve made with DBS controls of known parasite density.^51^^,^^52^ DNA concentrations from RDT samples were expressed in DBS parasite density equivalents, without adjustments for differences in DNA input between sample types. Only DBS samples were used to compare parasite densities across populations. All samples with >100 parasites/*µ*L of blood and a random selection of samples with parasitemia between 10 and 100 parasites/*µ*L of blood were selected for sequencing.

Amplicon sequencing libraries were prepared using the MAD4HatTeR targeted amplicon sequencing panel primer pools D1.1, R1.2, and R2.1.^53^ Libraries were sequenced on a NextSeq 2000 instrument (Illumina, San Diego, CA) using 150-cycle paired-end reads. A custom bioinformatics pipeline was used to identify alleles at 241 targets.^53^ This report includes SNPs and microhaplotypes (SNPs within the same amplicon) from the following genes: *kelch13*, *crt*, *mdr1*, *mdr2*, *dhps*, *dhfr*, *coronin*, apicoplast ribosomal protein S10, putative phosphoinositide-binding protein, ferrodoxin, exonuclease, and PF3D7_1322700. Lactate dehydrogenase gene target sequences in *P*. *falciparum*, *Plasmodium vivax* (*P*. *vivax*), *Plasmodium malariae*, *Plasmodium ovale* (*P*. *ovale*), and *Plasmodium knowlesi* were used to identify the presence of *Plasmodium* species in the samples. Targets in and around the *hrp2* and *3* genes were used to identify gene deletions. Microhaplotype sequences from 165 highly heterozygous targets were used to identify the complexity of infection (COI) and genetic relatedness. Variations in the original protocol and bioinformatics pipeline are described in Eloff, Aranda-Díaz et al, 2025.^54^

### Data cleaning, analysis, and visualization.

Data cleaning, analysis, and visualization were conducted in R (University of Auckland, New Zealand, version 4.3.1 [R Foundation, Vienna, Austria]). Samples with <10,000 reads, targets with <50 reads, and SNPs with within-sample allele frequency (WSAF) values <0.01 or with ≤10 reads were excluded from the analysis. Single-nucleotide polymorphisms observed in only one sample with WSAF <1, and SNPs observed in multiple samples but exclusively in a single sequencing run, were also excluded. Contaminations were identified in positive controls (cultured strains) using unexpected alleles relative to reference genomes, and in negative controls via the presence of reads. Batches with more than one control exhibiting contamination (>100 reads in >10 targets) were excluded.

Non-*falciparum* targets were excluded from sequencing depth summaries. Otherwise, all resulting data were analyzed, including samples with partial SNP coverage, resulting in variable sample counts for each assessed SNP. Samples were considered successfully sequenced if a genotype was obtained for all 59 SNPs evaluated in the *kelch13* gene. Multilocus haplotypes for polymorphisms at five independent targets in the *dhps* and *dhfr* genes (*dhps*: 436, 437, 540, 581, and 613; *dhfr*: 51, 59, 108, and 164) were identified for each sample by combining all individual microhaplotypes. Haplotypes were identified for samples with either unmixed genotypes at all targets or a single target showing a mixed genotype; in the latter case, the sample was assumed to carry the two haplotypes, accounting for the observed mixture. Samples with mixed genotypes in more than two amplicons were considered indeterminate.

To assess data patterns and identify potential issues with sampling and library preparation, sample similarity was estimated using the root mean square error of WSAF values for highly diverse loci (pool D1.1 targets), followed by density-based spatial clustering of applications with noise (ε = 0.2). Samples with clonality values ≥2 and nearly identical WSAF values across all loci, suggesting contamination or sample duplication, were excluded from the analysis.

## STATISTICAL ANALYSES

Descriptive statistics for sex, age group (<5, 5–14, 15–24, 25–39, 40–59, and >59 years), occupation (including the self-defined occupation category “minor”), overnight travel during the past 4 weeks, nationality, and method of detection were calculated. Individual ages were rounded up. For the analysis, samples were considered RDT-positive if they were positive for Pf only or for both Pf and *Pan*. Demographic differences between all RDT-positive samples and those successfully sequenced, as well as between administrative regions, were assessed using χ^2^ tests.

The proportion of samples that carried a given mutation or haplotype was calculated as the number of mixed and pure mutant genotypes divided by the total number of genotyped infections. The R function binom.test was used to estimate 95% CIs for all prevalences.

The COI for each sample and population allele frequencies were jointly estimated for each municipality and province using MOIRE (v3.4.0, EPPIcenter, San Francisco, CA), which implements a Markov chain Monte Carlo-based approach to identify the sample COI, within-host relatedness, and population allele frequencies from polyallelic genomic data.^55^ By modeling the genetic data and accounting for experimental errors, MOIRE provides probabilistic estimates. Microhaplotypes across all highly diverse *P. falciparum* targets (primer pool D1.1), as well as SNPs or microhaplotypes in drug-resistance targets of interest, were used to identify the COI and allele frequencies. The percentage of polyclonal infections was estimated as the mean of individual probabilities of polyclonality.

Univariate and multivariate logistic regression were performed to assess associations among the binary outcomes, presence of markers of interest, and polyclonality (probability of polyclonality >0.5), and key categorical variables, including age group, sex, occupation, detection method, and travel history. Analyses were restricted to mutations and variables with a sufficient sample size to achieve model convergence. Data from participants who reported travel to multiple countries were excluded from this analysis because a single potential country of origin could not be assigned.

Non-*falciparum* species were considered present if the combined reads across all *ldh* targets exceeded 100, with each species contributing more than 1% of the total *ldh* target reads.

Putative *hrp2* and *hrp3* deletions were identified by applying a generalized additive model to account for target length amplification bias and differences in depth of coverage across primer pools, as previously described.^53^ Read depth requirements for analysis were determined according to specifications in the original method publication.^53^ The model was fit on all data from the sequencing runs that included samples from the present study, as well as samples from other countries in the GenE8 project that were sequenced in the same runs. To account for intrinsic target biases not explained by target length variation, a correction factor for targets of interest was obtained from the median residuals of all field samples and controls known not to have a deletion in each sequencing run. Read depth fold changes were estimated for *hrp2* and *hrp3*, and samples were labeled as *hpr2*- or *hrp3*-deleted if the estimated fold change was <0.5. The workflow was validated using data from DBS controls generated with the cultured *P*. *falciparum* strain Dd2, which has an *hrp2* deletion and was included in all sequencing runs.

To characterize genetic relatedness between samples, an infection-level relatedness estimate ($\hat{r}$) based on identity-by-descent (IBD) was calculated for each sample pair using the *Dcifer* package and 165 highly heterozygous targets.^56^ The resulting $\hat{r}$ value is an aggregate of the IBD between clones in the two infections in the analyzed pair. For this analysis, population allele frequencies were estimated using maximum likelihood via *Dcifer*’s *calcAFreq* function, and the COI was estimated naively as the 95th quantile of the number of alleles observed at each target. Only samples with >75% coverage across all targets were included in the analysis. Sample pairs were considered related if the null hypothesis that $\hat{r}$ = 1 failed to be rejected at the 0.05 level. No correction was applied for multiple hypothesis testing because individual results are of less interest than population-level patterns. Clusters of related samples were visualized by creating networks with samples as nodes and links drawn between nodes if the two samples were considered related, as defined above.

## RESULTS

### Sample characteristics.

A total of 748 samples, comprising 713 DBS and 35 RDTs, along with the relevant survey data, were collected from 80 health facilities, beginning during the 2023 transmission peak (Figure 1A and B). Of these, 538 samples (504 DBS and 34 RDT) were collected from RDT-positive individuals, predominantly between April and June 2023 (Supplemental Figure 1A). Most of the 210 RDT-negative samples were collected during the second half of 2023. Parasite density was estimated for 741 of the 748 samples. Among qPCR-confirmed RDT-positive samples, parasite DNA content was higher in DBS extracts compared with RDT extracts (*P* <0.001; Supplemental Figure 1B). Parasite densities ≥100 parasites/*µ*L were observed in 83.4% of RDT-positive samples (439/526), 5.4% of RDT-negative samples (11/204), and two of the three Pf-negative/*Pan*-positive samples (Supplemental Figure 1C). The majority of RDT-negative samples (65.7%) had <1 parasite/*µ*L or undetectable parasite densities, with only 28.9% in the 1–100 parasite/*µ*L range.

Sequencing libraries were prepared for 461 samples with parasite densities ≥100 parasites/*µ*L and for an additional 20 samples with parasite densities between 10 and 100 parasites/*µ*L, representing 60 health facilities. Potential cross-contamination led to the exclusion of 19 samples with parasite densities ≥100 parasites/*µ*L and one sample with a parasite density between 10 and 100 parasites/*µ*L. Four samples with <10,000 total reads were excluded. The median per-sample sequencing depth was 552,825 reads (interquartile range [IQR]: 253,194–1,563,407), and the median per-target depth was 1,260 reads (IQR: 278–4,127). Sample sequencing depth was modestly correlated with parasite density (*R^2^* = 0.2; *P* <0.001; Supplemental Figure 2A). A median of 222 of 238 targets per sample (93.3%; IQR: 198–225) had >100 reads. Markers with the lowest genotyping success rates included *crt* 218/220, *mdr1* 86, and PF3D7_1322700 236, which were successfully genotyped in 98, 351, and 357 of the 457 samples, respectively, consistent with amplification biases reported in the original method publication.^53^ Of the 457 samples analyzed, 392 (85.7%) had a genotype in all 59 *kelch13* SNPs assessed.

There were no significant demographic or epidemiological differences among all RDT-positive participants and those whose samples were successfully sequenced (Supplemental Table 1). Most sequenced samples originated from the Hhohho (51%) and Lubombo (36%) Regions (Table 1), consistent with malaria incidence in those regions (Figure 1B). Participants were predominantly male (78%) and aged 15–39 years (68%). Nearly half (46%) of samples were collected from agricultural workers, who accounted for 63% of cases in the Hhohho Region. Swazi nationals comprised the majority of participants with successfully sequenced samples (80%), followed by Mozambican nationals (20%). Most cases (93%) were detected through passive surveillance, and in the Hhohho Region, 98% were detected passively. Of the sequenced samples, 25% were collected from participants who reported travel to Mozambique, and 7.5% were collected from those who reported travel within Eswatini. Compared with non-travelers, individuals who had traveled to Mozambique were more likely to be male, agricultural workers, and detected in the Manzini Region (Supplemental Table 2). No significant differences in parasite densities were observed among regions for successfully sequenced samples (*P* >0.4; Mann–Whitney *U* test).

**Table 1:** Characteristics of study participants with successfully sequenced samples

Characteristic	Overall	Hhohho	Lubombo	Manzini	Shiselweni
Health facilities	54	17	22	12	3
*N*	392	200	140	45	7
Sex					
Female	85 (22%)	29 (15%)	38 (27%)	17 (38%)	2 (29%)
Male	305 (78%)	171 (86%)	102 (73%)	28 (62%)	5 (71%)
Age (years)					
<5	12 (3.1%)	3 (1.5%)	7 (5.1%)	2 (4.5%)	0 (0%)
5–14	50 (13%)	19 (9.5%)	22 (16%)	7 (16%)	2 (29%)
15–24	134 (35%)	74 (37%)	46 (34%)	11 (25%)	3 (43%)
25–39	127 (33%)	81 (41%)	40 (29%)	6 (14%)	0 (0%)
40–59	48 (12%)	17 (8.5%)	16 (12%)	13 (30%)	2 (29%)
>59	16 (4.1%)	5 (2.5%)	6 (4.4%)	5 (12%)	0 (0%)
Unknown	5	1	3	1	0
Occupation					
Minor	16 (4.1%)	4 (2.0%)	9 (6.5%)	3 (6.8%)	0 (0%)
Student	84 (22%)	30 (15%)	40 (29%)	10 (23%)	4 (57%)
Agricultural	179 (46%)	125 (63%)	51 (37%)	3 (6.8%)	0 (0%)
Other	41 (11%)	15 (7.5%)	6 (4.3%)	18 (41%)	2 (29%)
Unemployed	69 (18%)	26 (13%)	32 (23%)	10 (23%)	1 (14%)
Unknown	3	0	2	1	0
Nationality					
Eswatini	312 (80%)	157 (79%)	122 (88%)	27 (61%)	6 (86%)
Mozambique	78 (20%)	43 (22%)	17 (12%)	17 (39%)	1 (14%)
Unknown	2	0	1	1	0
Travel					
No travel	253 (65%)	140 (70%)	94 (67%)	15 (34%)	4 (57%)
Domestic	29 (7.4%)	7 (3.5%)	16 (11%)	6 (14%)	0 (0%)
Mozambique	98 (25%)	49 (25%)	27 (19%)	21 (48%)	1 (14%)
Other countries	9 (2.3%)	2 (1.0%)	3 (2.1%)	2 (4.5%)	2 (29%)
Multiple countries	2 (0.5%)	2 (1.0%)	0 (0%)	0 (0%)	0 (0%)
Unknown	1	0	0	1	0
Detection method					
Passive	364 (93%)	196 (98%)	119 (86%)	43 (98%)	6 (86%)
Reactive	24 (6.2%)	4 (2.0%)	18 (13%)	1 (2.3%)	1 (14%)
Active	2 (0.5%)	0 (0%)	2 (1.4%)	0 (0%)	0 (0%)
Unknown	2	0	1	1	0
Parasite density (parasite/*µ*L)*					
*n*	421	215	153	46	7
Median (IQR)	83,506 (14,498–373,994)	77,502 (19,203–275,072)	80,458 (11,645–391,803)	92,366 (10,695–573,685)	721,667 (72,238–28,725,781)

IQR = interquartile range.

* Samples with partial sequencing coverage were included in parasite density analysis.

### Genomics reveals low levels of antimalarial drug resistance markers of concern.

Among the 443 samples with adequate sequencing depth for *hrp2*/*3* deletion analysis, only three were from RDT-negative individuals. No evidence of the *hrp2*/*3* gene deletions was detected in any sample. Non-*falciparum* coinfections were rare: *Plasmodium malariae* was detected in only three samples (0.7%), including two from the Lubombo Region and one from the Hhohho Region. These included one Swazi and two Mozambican nationals who reported travel to Mozambique. No coinfections with *P*. *ovale* or *P*. *vivax* were observed.

Overall, 61.4% of samples (95% CI: 59.9–63.2%) were polyclonal, with a mean COI of 2.54 (95% CI: 2.48–2.61). The proportion of polyclonal infections and mean COI were significantly lower in the Hhohho Region compared with the other three regions (Table 2). Participants who reported travel to Mozambique were twice as likely to carry polyclonal infections (odds ratio [OR]: 2.51; 95% CI: 1.44–4.44; Supplemental Table 3). In contrast, samples with higher parasitemia (OR: 0.71; 95% CI: 0.59–0.86; Supplemental Figure 2B) and higher sequencing depth (OR: 0.58; 95% CI: 0.38–0.86) were less likely to be polyclonal. No significant associations were observed between polyclonality and participant demographics. Mixed genotypes in at least one drug resistance marker were detected in 39.3% of the samples.

**Table 2:** Complexity of infection and percentage of polyclonal infections for each administrative region in Eswatini

	Overall	Hhohho	Lubombo	Manzini	Shiselweni
*N*	456	238	162	49	7
Mean COI (95% CI)	2.54 (2.48–2.61)	2.37 (2.29–2.45)	2.72 (2.62–2.82)	2.67 (2.51–2.88)	3.61 (3.14–4.42)
Percentage polyclonal (95% CI)	61.4% (59.9–63.2%)	55.6% (53.4–58.4%)	65.1% (61.1–68.5%)	74.1% (69.4–77.6%)	85.4% (71.4–100%)

COI = complexity of infection. CIs were drawn from posterior distributions.

### Mutations in the *dhps* and *dhfr* genes.

Mutations in the *dhps* and *dhfr* genes, which are associated with resistance to SP, were highly prevalent (Supplemental Table 4). The *dhps* ISGEAA double mutant haplotype (mutations A437G and K540E; wild type at I431, S436, A581, and A613) was detected in 94.4% of the samples (*N* = 429), with the highest prevalence in the Hhohho Region (96.4%; *N* = 225; Supplemental Table 4). The ISGEGA triple mutant haplotype (carrying an additional A581G mutation) was observed in 6.8% of samples overall, reaching 12.8% (*N* = 47) in the Manzini Region. Wild-type (*dhps* ISAKAA) and the I431V-containing variant (VAGKGS) were rare, each observed in <3% of samples. No *dhps* mutant haplotypes ISGEGS or ISGEAS were detected. Haplotype inference was not possible for 22 samples (5.1%). The *dhfr* IRNI triple-mutant haplotype (mutations N51I, C59R, and S108N; wild-type I164) was nearly fixed, present in 98.9% of samples (*N* = 453; Supplemental Table 4). A single sample (0.2%) carried the wild-type NCSI haplotype, with haplotype inference not possible for one sample. The I164L mutation was not detected in any of the samples analyzed. Among the 429 samples with complete *dhps*/*dhfr* haplotypes (Table 3), 93.2% carried the quintuple mutant haplotype (*dhps* ISGEAA and *dhfr* IRNI), whereas 6.8% carried the sextuple mutant haplotype (*dhps* ISGEGA and *dhfr* IRNI). All non-ISGEAA *dhps* haplotypes were found in combination with the *dhfr* IRNI haplotype.

**Table 3:** Proportion of samples carrying antimalarial drug resistance markers in Eswatini

	Overall		Hhohho		Lubombo		Manzini		Shiselweni	
*dhps dhfr*	*n*/*N*	% (95% CI)	*n*/*N*	% (95% CI)	*n*/*N*	% (95% CI)	*n*/*N*	% (95% CI)	*n*/*N*	% (95% CI)
ISAKAA IRNI (wild type)	10/429	2.3% (1.1–4.2%)	5/227	2.2% (0.7–5.1%)	4/149	2.7% (0.7–6.7%)	0/47	0%	1/6	16.7% (0.4–64.1%)
ISGKAA IRNI	8/429	1.9% (0.8–3.6%)	2/227	0.9% (0.1–3.1%)	3/149	2.0% (0.4–5.8%)	3/47	6.4% (1.3–17.5%)	0/6	0% (0–45.9%)
ISAEAA IRNI	3/429	0.7% (0.1–2%)	0/227	0% (0–1.6%)	2/149	1.3% (0.2–4.8%)	1/47	2.1% (0.1–11.3%)	0/6	0% (0–45.9%)
ISGEAA NCSI	1/429	0.2% (0–1.3%)	1/227	0.4% (0–2.4%)	0/149	0% (0–2.4%)	0/47	0% (0–7.5%)	0/6	0% (0–45.9%)
ISGEAA NRNI	18/429	4.2% (2.5–6.6%)	5/227	2.2% (0.7–5.1%)	11/149	7.4% (3.7–12.8%)	2/47	4.3% (0.5–14.5%)	0/6	0% (1.8–42.8%)
ISGEAA IRNI (quintuple)	400/429	93.2% (90.4–95.4%)	216/227	95.1% (91.5–97.6%)	138/149	92.6% (87.2–96.3%)	42/47	89.4% (76.9–96.5%)	4/6	66.7% (22.3–95.7%)
ISGEGA IRNI (sextuple)	29/429	6.8% (4.6–9.6%)	10/227	4.4% (2.1–8.0%)	13/149	8.7% (4.7–14.5%)	6/47	12.8% (4.8–25.7%)	0/6	0% (0–45.9%)
VAGKGS IRNI	3/429	0.7% (0.1–2%)	1/227	0.4% (0–2.4%)	0/149	0% (0–2.4%)	1/47	2.1% (0.1–11.3%)	1/6	16.7% (0.4–64.1%)
Undetermined*	22	–	6	–	13	–	2	–	1	–
*crt*										
F48L	12/446	2.7% (1.4–4.7%)	10/232	4.3% (2.1–7.8%)	2/159	1.3% (0.2–4.5%)	0/48	0% (0–7.4%)	0/7	0% (0–41.0%)
72–76 CVIET	2/457	0.4% (0.1–1.6%)	2/238	0.8% (0.1–3.0%)	0/162	0% (0–2.3%)	0/50	0% (0–713%)	0/7	0% (0–41.0%)
A220S	5/99	5.1% (1.7–11.4%)	5/50	10.0% (3.3–21.8%)	0/38	0% (0–9.3%)	0/8	0% (0–36.9%)	0/3	0% (0–70.8%)
*k13*										
P553L	4/431	0.9% (0.3–2.4%)	1/222	0.5% (0.0–2.5%)	3/156	1.9% (0.4–5.5%)	0/46	0% (0–7.7%)	0/7	0% (0–41.0%)
*mdr1*										
F184Y	254/440	57.7% (53.0–62.4%)	105/228	46.1% (39.5–52.8%)	115/158	72.8% (65.1–79.6%)	28/47	59.6% (44.3–73.6%)	6/7	85.7% (42.1–99.6%)
*mdr2*										
I492V	100/392	25.5% (21.3–30.1%)	49/206	23.8% (18.1–30.2%)	33/138	23.9% (17.1–31.9%)	14/41	34.1% (20.1–50.6%)	4/7	57.2% (18.4–90.1%)

crt = chloroquine resistance transporter; dhfr = dihydrofolate reductase; dhps = dihydropteroate synthase; mdr = multidrug resistance.

* Undetermined polyclonal samples.

Multivariate analysis indicated that participants who had recently traveled internationally to countries other than Mozambique were less likely to carry the quintuple mutant haplotype, although the sample size was small (OR: 0.05; 95% CI: 0.01–0.23; Supplemental Table 5). All infections carrying the sextuple mutant haplotype were detected through passive surveillance between April and May 2023 (Figure 2), coinciding with the peak in sample collection (Figure 1C). No association with sex, age, occupation, or parasitemia was found for either the quintuple or the sextuple mutant haplotypes (Supplemental Tables 5 and 6). The three samples that carried the *dhps* VAGKGS haplotype were monoclonal and geographically dispersed. Of those, two were collected in the Hhohho and Shiselweni Regions from patients who reported recent travel to Tanzania. Both presented to health facilities within 3 days of each other in July 2023 (Figure 2). The third, from Manzini, was a participant with no reported travel history who presented to the health facility 44 days later.

**Figure 2.**
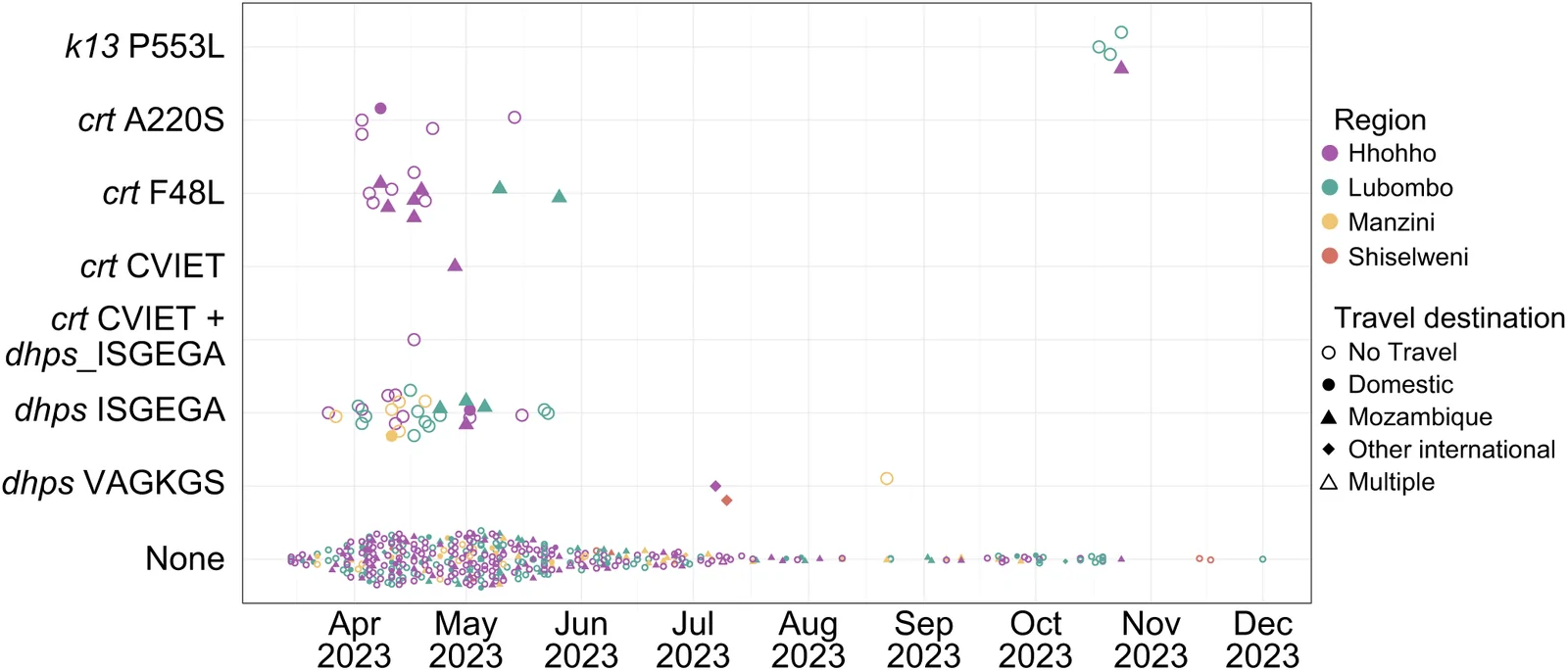
Temporal distribution of samples carrying genotypes of interest. Collection date and region, genotype, and travel history for each sample in the dataset are indicated.

### Mutations in the *crt* gene.

The *crt* 72–76 CVIET mutant haplotype, which is associated with chloroquine resistance, was detected in only two of 457 samples (0.4%; 95% CI: 0.1–1.6%), both from the Hhohho Region. These samples were polyclonal and carried mixed *crt* 72–76 mutant CVIET and wild-type CVMNK haplotypes. The *crt* A220S mutation was found in five of 99 samples with genotype data for this locus (5.1%; 95% CI: 1.7–11.4%), all from the Hhohho Region, representing 10% of samples sequenced in that region. All A220S mutations co-occurred with the wild-type *crt* 72–76 CVMNK haplotype. The *crt* F48L mutation was detected in 12 samples (2.7%; 95% CI: 1.4–4.7%), including 10 from the Hhohho Region (4.3%; 95% CI: 2.1–7.9%) and two from the Lubombo Region (1.3%; 95% CI: 0.2–4.5%). These also occurred exclusively with the wild-type *crt* 72–76 CVMNK haplotype. All *crt* mutations were found in samples collected between April and May 2023, in both travelers and non-travelers (Figure 2). The *crt* I356T mutation was not observed.

### Mutations in the kelch13 gene.

The only *kelch13* propeller domain mutation identified was P553L, a validated marker of ART-R. It was detected in four of 456 samples (0.9%; 95% CI: 0.3–2.4%): one from the Hhohho Region (0.5%; 95% CI: 0.0–2.5%) and three from the Lubombo Region (1.9%; 95% CI: 0.4–5.5%). All four samples were collected during the second half of October 2023 (Figure 2), and three were polyclonal. One case, from the Hhohho Region, involved a traveler to Mozambique; the rest of the patients reported no travel. Of the P553L-positive individuals, three were agricultural workers, and one was a minor.

### Mutations in the *mdr1* and *mdr2* genes.

The *mdr1* N86 wild-type genotype was found in all 351 samples with a valid genotype in that locus. No samples carried the *mdr1* N86Y genotype. In contrast, the *mdr1* F184Y genotype was detected in 57.6% of the samples (95% CI: 52.9–62.3%), with regional prevalence ranging from 39.5% in the Hhohho Region (95% CI: 39.5–52.8%) to 85.7% in the Shiselweni Region (95% CI: 42.1–99.6%; Table 3). This mutation was not significantly associated with patient characteristics (Supplemental Table 7). The *mdr2* I492V mutation was identified in 25.6% of the samples (95% CI: 21.3–30.2%), with similar prevalence across all four regions (Table 3). Like the *mdr1* F184Y mutation, this mutation was not significantly associated with demographic or clinical variables (Supplemental Table 8).

### Samples carrying mutations form clusters of highly related infections.

The IBD-based measure of infection-level genetic relatedness ($\hat{r}$) among samples was bimodal, with peaks at 0 and 1 (Supplemental Figure 3). Using a threshold of $\hat{r}$ = 1 as the null hypothesis, 235 of 405 samples (58.0%) were classified as highly related to at least one other sample, yielding 988 highly related sample pairs (1.21% of all sample pairs). Clustering analysis revealed widespread genetic clustering: six clusters contained more than five samples, whereas 36 additional clusters consisted of five or fewer samples (Figure 3A). Lowering the relatedness threshold to $\hat{r}$ > 0.5 increased the cluster size but did not change the overall cluster patterns (Supplemental Figure 4A). Polyclonality was significantly more frequent in samples not assigned to any cluster (61.8%) compared with those within clusters (41.3%; *P* <0.001; χ^2^ test). Similarly, unrelated samples were significantly more likely to come from participants who reported international travel (41.3% versus 17.2%; *P* <0.001; χ^2^ test). Clusters were typically composed of samples collected from the same region and similar time frames, although some spanned multiple months and regions (Figure 3B).

**Figure 3.**
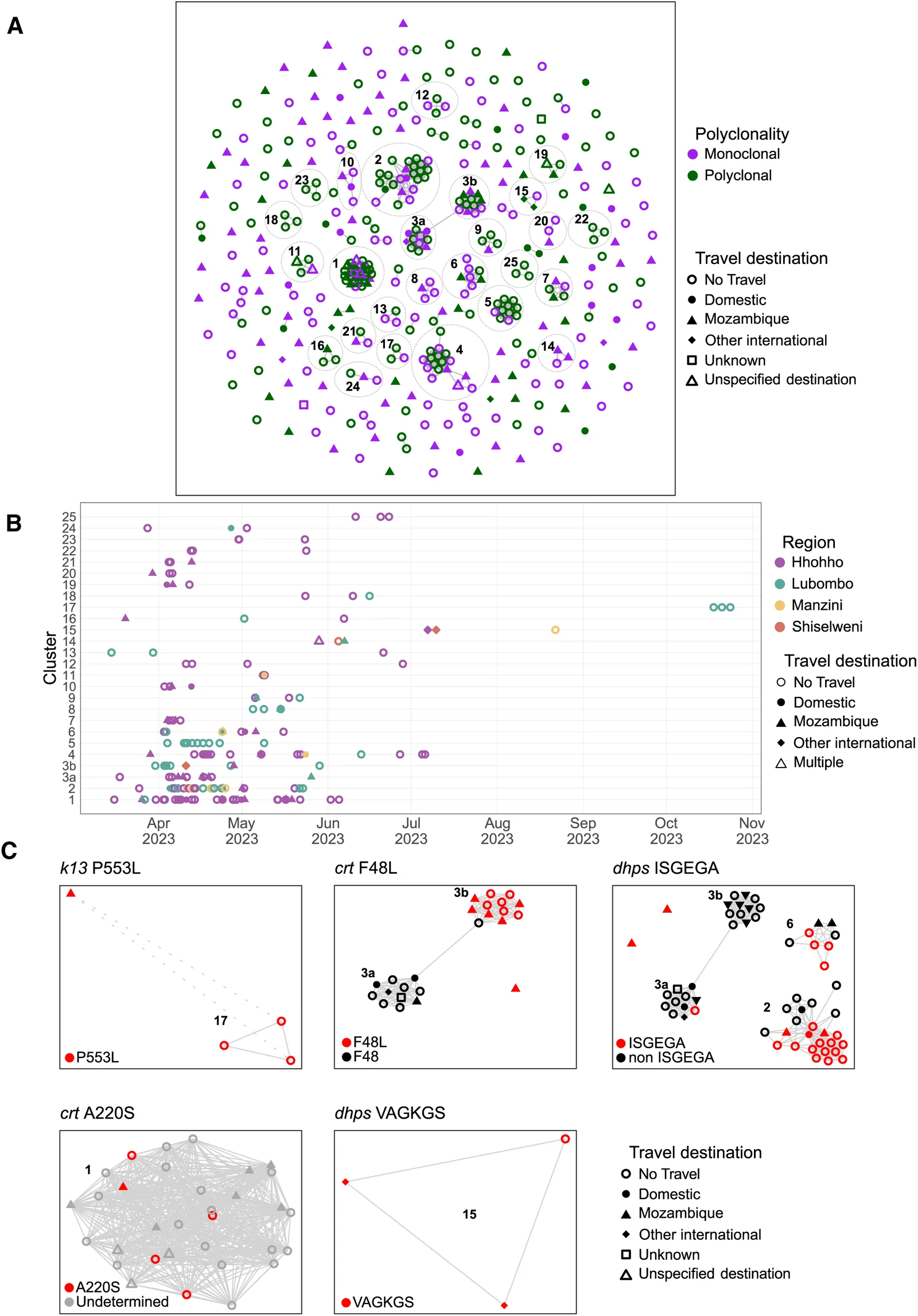
Genotypes of interest are segregated into genetically related clusters. (**A**) Clusters of genetically related samples, defined as sample pairs with a relatedness value of $\hat{r}$ = 1, at the 0.05 significance level. Only clusters composed of three or more samples are labeled and highlighted with a dashed outline. (**B**) Samples belonging to each cluster of three or more samples by collection date and region. (**C**) All clusters and isolated samples containing a genotype of interest are shown, indicating the genotype for each sample in those clusters. Cluster labels are the same as panels A and B. Missing genotypes due to low depth of coverage are indicated in gray. Dashed lines indicate significantly related pairs with a lower $\hat{r}$ value (0.5) only shown for samples carrying the *kelch13* P553L mutation.

Although mutations in the *dhps* (ISGEGA and VAGKGS haplotypes), *crt* (F48L, 72–76 CVIET and A220S), and *kelch13* (P553L) genes were detected at low prevalence, they were often observed in temporally clustered samples and, in some cases, within close geographic proximity. These mutations of interest were mostly segregated within related clusters (Figure 3C). Of the four samples that carried *kelch13* P553L, three were in cluster 17, whereas the fourth exhibited significant relatedness to samples in that cluster when the threshold used for hypothesis testing was lowered to 0.5 (Supplemental Figure 4B). All but one sample that carried the *crt* F48L mutation belonged to cluster 3, which contained 23 samples organized into two tightly connected subclusters (3a and 3b) that overlapped in time and were linked by polyclonal infections. The F48L mutation was restricted to subcluster 3b. These subclusters included samples from multiple individuals who had traveled to Mozambique. All samples with a valid A220S mutation were detected exclusively in cluster 1. All other samples in this cluster lacked genotype calls for amino acid 220, and none were successfully classified as wild type. Cluster 1 was predominantly composed of samples collected in the Hhohho Region, with several from participants who reported travel to Mozambique. All three samples that carried the *dhps* VAGKGS haplotype were found in cluster 15. The *dhps* ISGEGA haplotype was distributed across three mixed *dhps* genotype clusters (clusters 2, 3a, and 6) and also appeared in two unclustered samples from travelers to Mozambique. Cluster 2 included the majority of samples with *dhps* ISGEGA, including several tightly connected samples from non-travelers and two from travelers to Mozambique. These mutations (*dhps* ISGEGA or VAGKGS; *crt* F48L, CVIET or A220S; or *k13* P553L) were present in 11.9% of all samples and were more common in larger clusters. Although 25.2% of all samples were part of clusters with more than five samples, 58.3% of the samples that carried at least one of these mutations belonged to such clusters.

## DISCUSSION

The present malaria molecular surveillance study, which involves targeted amplicon sequencing, provides the first data on antimalarial drug and diagnostic resistance markers from Eswatini since 2010. In addition to the assessment of marker prevalence, parasite genomic data were used to characterize population structure and transmission dynamics, providing critical insights into the emergence and spread of drug-resistant parasites in low-endemic settings. The validated ART-R marker, *kelch13* P553L, was detected at low prevalence but formed a genetically distinct cluster of highly related infections in the Hhohho and Lubombo Regions. Although the *crt* K76T chloroquine resistance marker was nearly absent, SP resistance markers were nearly fixed: 95% of samples carried the quintuple mutant haplotype (*dhps* ISGEAA + *dhfr* IRNI), and 7% carried the sextuple mutant haplotype (*dhps* ISGEGA + *dhfr* IRNI). These haplotypes were concentrated in transmission clusters, supporting the role of local amplification. The *mdr1* N86 wild-type genotype, which is associated with reduced lumefantrine susceptibility, was present in all samples.^19^ Non-*falciparum* species were rare, and no *hrp2*/*3* deletions were identified.

Eswatini exclusively relies on artemether–lumefantrine for the treatment of uncomplicated malaria. The rapid emergence and spread of ART-R in East Africa and the Horn of Africa, along with recent reports of reduced lumefantrine susceptibility and decreased artemether–lumefantrine efficacy, pose a growing risk of the de novo emergence or introduction of ART-R in Eswatini.^6^^,^^9^^,^^57^^,^^58^ The detection of the validated ART-R marker *kelch13* P553L in a localized cluster, including one case linked to travel from Mozambique and others with no associated travel history, raises concern for both importation and onward transmission. This mutation has been associated with delayed clearance in Southeast Asia but has been rarely reported in sub-Saharan Africa.^12^^,^^59^^–^^61^ Notably, this same mutation was recently reported in South Africa’s Mpumalanga Province, which borders Eswatini to the north and west, in October 2023.^62^ Whether parasites carrying this mutation persisted into the 2023/2024 transmission season remains unknown. Additional research is critical to assess the clinical impact of these parasites in southern Africa and to determine their ancestral origins. No other *kelch13* mutations identified in other African countries were detected in the present study.^6^^,^^54^^,^^63^^,^^64^

Even in the presence of ART-R, ACTs are expected to remain effective because of the action of partner drugs. Although reduced lumefantrine susceptibility has been reported in Uganda, no full resistance has been documented, and no validated genetic markers of lumefantrine resistance currently exist.^6^^,^^57^ The *mdr1* N86 genotype, which is associated with a modest reduction in susceptibility in laboratory studies and has been shown to be selected for by artemether–lumefantrine use, was found in all samples in the present study, possibly reflecting the selective pressure of the drug.^7^^,^^57^^,^^65^ Molecular markers of resistance to other ACT partner drugs, such as mutations in *crt* and *mdr1* associated with piperaquine and amodiaquine resistance, were not detected.^18^^,^^19^ Duplications of *mdr1* or *plasmepsin 2*/*3* linked to mefloquine and piperaquine resistance, respectively, were not assessed.^20^^,^^21^

Mutations conferring resistance to former first-line antimalarials, including chloroquine and SP, exhibited patterns consistent with current drug use and malaria epidemiology in the region. The chloroquine resistance-associated *crt* CVIET haplotype, previously widespread under chloroquine pressure, was nearly absent.^36^ The low-prevalence *crt* A220S and F48L mutations require further investigation. The A220S mutation can confer higher levels of chloroquine resistance when the *crt* CVIET haplotype is present.^66^ In the current study, only one-quarter of the samples had a valid genotype at *crt* 220 because of low amplification efficiency, and all samples with the A220S mutation were found in conjunction with the wild-type *crt* CVMNK haplotype. The *crt* F48L mutation has not been linked to resistance to any antimalarials, but it has been detected at low levels in southern Africa.^64^ Sulfadoxine–pyrimethamine has not been used for treatment in Eswatini since the adoption of artemether–lumefantrine in 2010, but it remains widely used for chemoprophylaxis across sub-Saharan Africa.^67^ The high prevalence of the *dhps*/*dhfr* quintuple mutant haplotype in Eswatini likely reflects importation from Mozambique, where SP is routinely used in intermittent preventive treatment in pregnancy.^68^ The sextuple mutant haplotype, which is associated with higher SP resistance, was found in 7% of samples, higher than the prevalence recently reported in Mozambique and South Africa (<1%).^62^^,^^68^ A cluster of three samples carrying the *dhps* VAGKGS haplotype was detected. This haplotype has been reported primarily in West and Central Africa and seems to be selected for by SP use in pregnancy.^69^^,^^70^

Genetic relatedness analysis revealed substantial clustering among infections, with a large proportion of samples belonging to highly related clusters, which frequently included samples carrying key resistance markers. More than 70% of patients reported no recent international travel, highlighting the risk of local amplification of resistant strains in low-transmission settings such as Eswatini. The generated genetic data, combined with travel histories for samples carrying the *kelch13* P553L mutation, which is a validated marker of ART-R, do not clarify the linkage between these cases. The infection in the traveler to Mozambique could have originated from the three locally acquired cases if the traveler had remained gametocyte-positive during the month between arrival and sample collection, or the parasite may have been related to a missed common ancestor of the locally acquired cases. De novo emergence of the mutation within Eswatini cannot be ruled out. Similarly, the *dhps* VAGKGS haplotype appears to have been co-imported by two travelers from Tanzania into two distinct locations in different regions, with a single linked secondary infection detected 6 weeks later in a third location, in a separate region. In contrast, clusters containing *crt* F48L or A220S mutations included separate travelers to Mozambique, suggesting that multiple introduction events may have seeded these clusters. Samples carrying the sextuple mutant haplotype ISGEGA were found in three large clusters, each of which included at least one traveler to Mozambique. These patterns suggest that the rapid spread of parasites contributed to the unexpectedly high prevalence of this marker in Eswatini. Overall, these results highlight how cross-border importation and local amplification can be interlinked processes that shape the epidemiology of antimalarial drug resistance, even in the presumptive absence of local drug-selective pressure. The rapid expansion of closely related parasite populations may facilitate the emergence of resistant genotypes in low-transmission settings. Further research is needed to identify fine-scale transmission networks to clarify pathways of parasite spread.

Higher malaria transmission intensity is typically associated with a greater COI.^39^^,^^71^^,^^72^ More than 60% of the samples in the present study were polyclonal. Although polyclonality was more common among travelers to Mozambique, likely reflecting the higher parasite diversity in source populations, more than half of infections among individuals who reported no international travel were also polyclonal. This suggests that ongoing local transmission contributes to superinfections and the cotransmission of multiple clones within transmission chains. The negative association between parasite density and COI may reflect host immune responses in a population with low immunity interacting with within-host parasite diversity or intra-host parasite interactions and warrants further investigation.

A previous study conducted in Eswatini among patients who presented to health facilities revealed that of 162 PCR-detectable infections, 33% were subpatent, and no *hrp2*/*3* deletions were identified.^32^ The majority of RDT-negative samples analyzed in the present study (both Pf- and *Pan*-negative) were PCR-negative or had low parasite density, and 28.1% had parasite densities in the 1–100 parasites/*µ*L range. Of the RDT-negative samples, 5% had parasite densities greater than 100 parasites/*µ*L, which could be due to mislabeling, database errors, or the prozone-like effect. The blood concentration of the HRP2 antigen was not measured in these samples. Among the RDT-negative and Pf-negative/*Pan*-positive samples with >100 parasites/*µ*L, only a subset had sufficient sequencing depth to assess for *hrp2*/*3* deletions, but no putative *hrp2*/*3* deletions were identified in either RDT-negative or RDT-positive samples. These findings, along with recent data from Mozambique and South Africa, suggest that there is no evidence for RDT misdiagnosis due to *hrp2*/*3* deletions.^62^^,^^73^ A recent study from South Africa’s KwaZulu-Natal Province, which borders Eswatini’s Hhohho and Shiselweni Regions, revealed a high prevalence of *hrp2*/*3* deletions using nested PCR testing; however, the small sample size, along with methodological and sample quality differences, may explain the discrepancy.^74^ Overall, the study data confirm that subpatent infections occur even in symptomatic cases identified at health facilities.

The present study had several limitations. Sample collection began at the peak of the 2022–2023 transmission season, missing the critical early-season travel period in December and January. As a result, early-season transmission dynamics could not be captured, and bias may have been introduced into prevalence estimates because of the temporal and spatial clustering of cases. Additionally, RDT-negative individuals were sampled in the second half of 2023, when malaria transmission and test positivity rates are typically lowest, further reducing the authors’ ability to detect and quantify *hrp2/3* deletions. Finally, asymptomatic infections and those occurring in hard-to-reach populations were less likely to be captured in this dataset because data were mostly collected through passive surveillance systems. Although all malaria cases within a single transmission season were targeted, the achieved sample size was smaller than anticipated because of the delayed start, which limited both the precision of prevalence estimates and the statistical power to detect associations between mutations and case characteristics. Approximately 15% of RDT-positive samples yielded incomplete sequencing results, which were evenly distributed across time and regions, and no sociodemographic differences were observed, reducing the likelihood of selection bias. Technical limitations of the sequencing assay further impacted data completeness. Uneven read depth across loci reduced the effective sample size for certain markers, particularly *crt* mutations. Additionally, phasing SNPs across amplicons to reconstruct haplotypes was not always possible.

## CONCLUSION

The revolution in genomic technologies has opened new avenues to study the adaptation and spread of malaria parasites within and across borders, offering valuable epidemiological insights for improving elimination strategies. Clusters of genetically related infections carrying markers of antimalarial drug resistance, including ART-R, were identified in the present genomic surveillance study. Although the overall prevalence of these markers was low, the findings support a pattern of introduction and local spread as the mechanism for the emergence of resistance in low-transmission settings. Therefore, routine molecular surveillance to track trends and deliver timely data is urgently needed to inform interventions to prevent the spread of resistant parasites in low-transmission areas.

## Supplemental Materials

10.4269/ajtmh.25-0487 Supplemental Materials

## Data Availability

Deidentified individual-level genotypes, COIs, and polyclonality probability data are available in the repository with the following DOI: 10.5281/zenodo.15387344.
