# Supplementary material for: Genomic Surveillance Reveals Clusters of Plasmodium falciparum Antimalarial Resistance Markers in Eswatini, A Low-Transmission Setting
Source: Am J Trop Med Hyg. 2026 May 19;115(5 Suppl):31–43. doi: 10.4269/ajtmh.25-0487 (PMC13737250; doi:10.4269/ajtmh.25-0487)
Supplement: Supplementary file 1 [file tpmd250487.SD1.pdf]

Supplemental Material

Genomic Surveillance Reveals Clusters of *Plasmodium falciparum* Antimalarial Resistance Markers in Eswatini, a Low-Transmission Setting

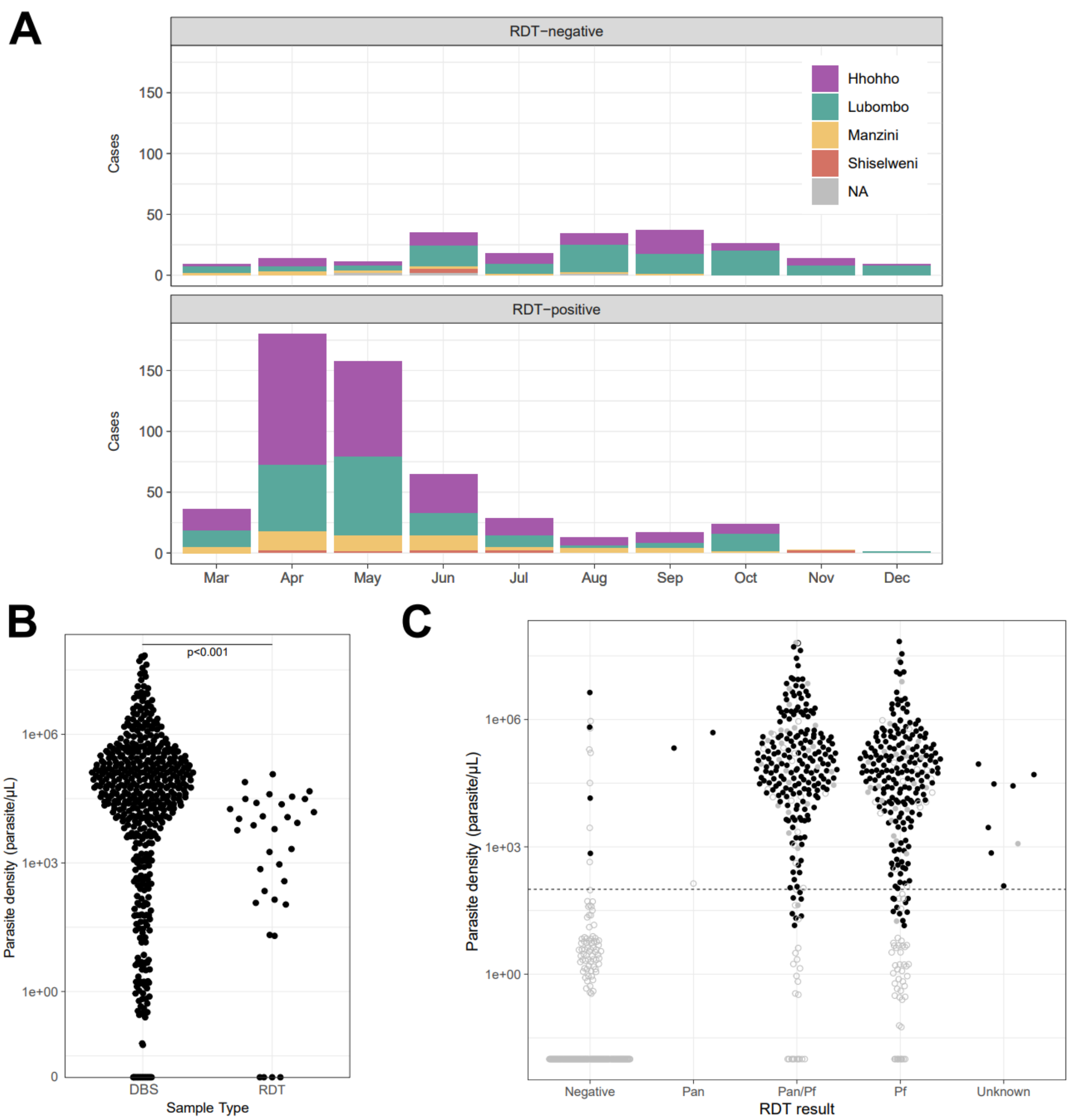

Figure S1. Sample RDT results and parasite density. A. Time of collection of RDT-positive (Pf or Pf/Pan RDT result) or RDT-negative (Negative or Pan-only) samples. **B.** Difference in parasite density in DNA extracts from DBS or RDT from RDT-positive samples. The reported *P*-value corresponds to a Wilcoxon rank-sum test. **C.** Parasite density in samples with different reported RDT results. Samples with a genotype in all 59 assessed *kelch13* SNPs are indicated in closed circles, and those missing at least one SNP in open circles. Samples with a valid *hrp2/3* target depth fold change estimate are indicated in black, and those without sufficient depth of coverage in gray. None of the samples with a valid *hrp2/3* target depth fold change were classified as *hrp2*- or *hrp3*-deleted. The dashed line indicates 100 parasites/μL.

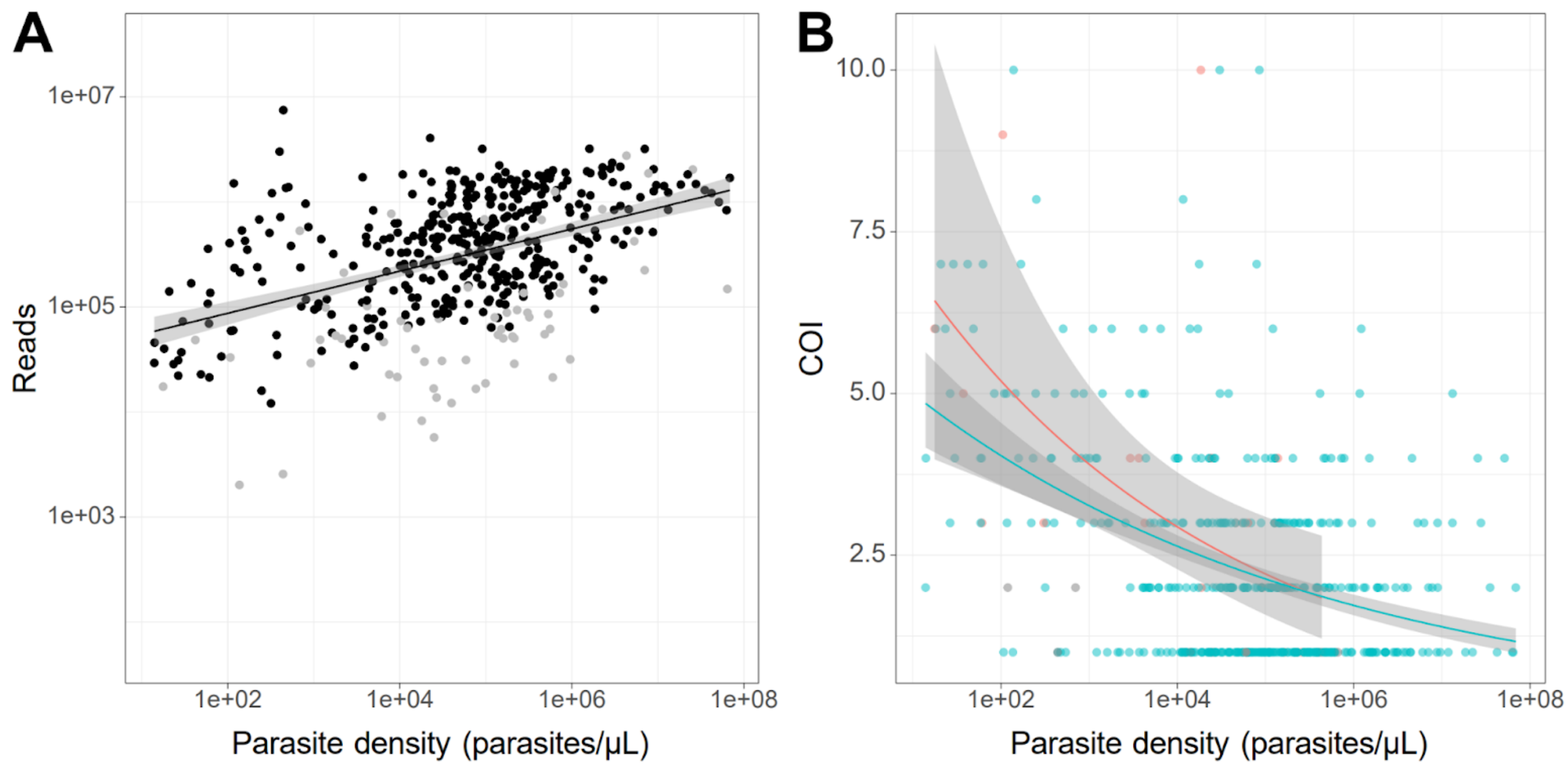

Figure S2. Relationship between parasite density, depth of coverage, and intrahost diversity. A. Linear regression of sample parasite density and total sample reads, for samples with a valid genotype in all 59 assessed *kelch13* SNPs (black) and those missing at least 1 valid SNP (grey). R^2=0.19, P <0.001. **B.** Quasi-Poisson regression of sample parasite density and complexity of infection (COI) for samples collected passively (cyan) or by proactive and reactive case detection (red). Grey samples are RDT-negative.

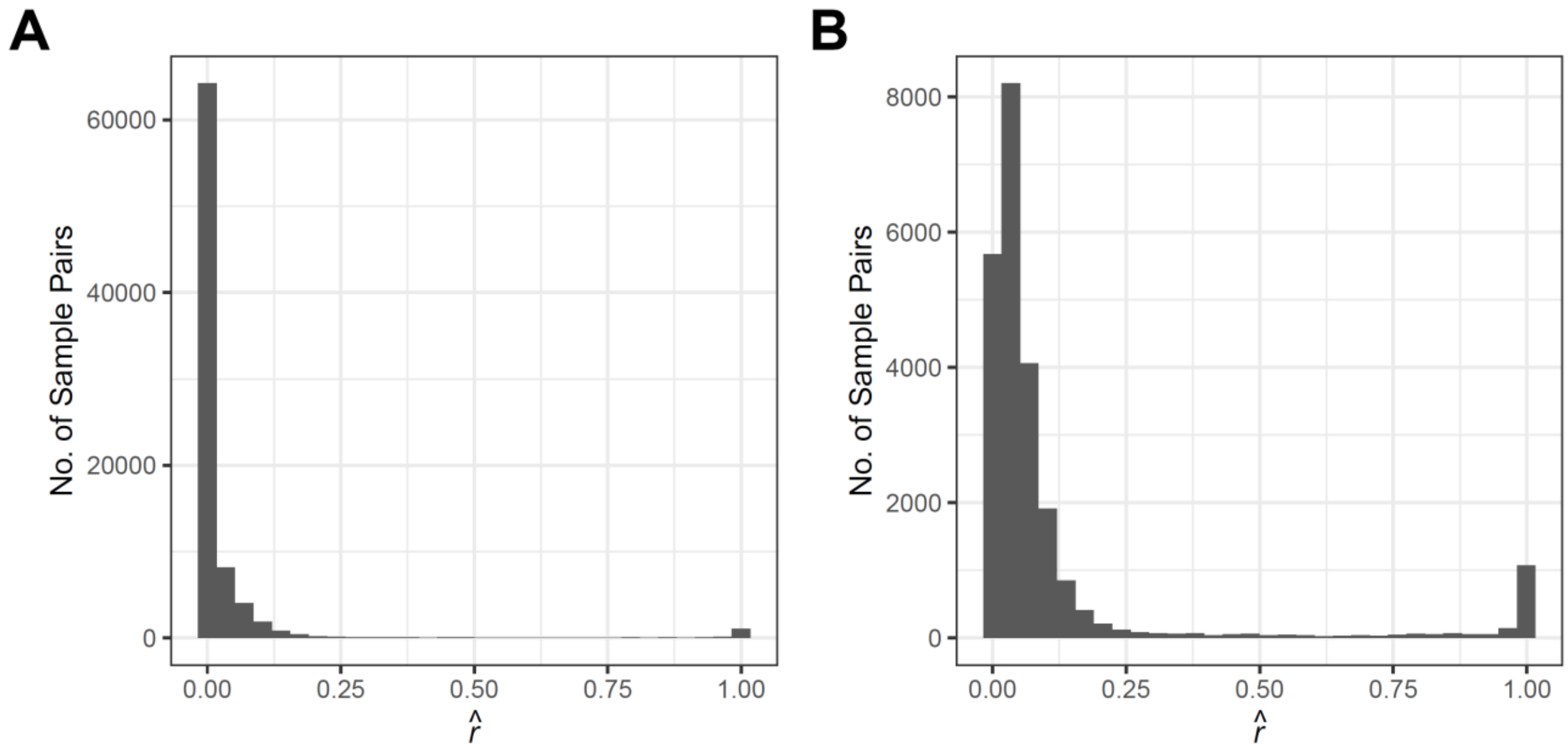

Figure S3: Distribution of estimated relatedness ($\hat{r}$) between all sample pairs. A. Histogram showing $\hat{r}$ among all pairs of samples in the dataset. **B.** As A, except pairs with an $\hat{r}$ of zero have been removed, to better visualize the remainder.

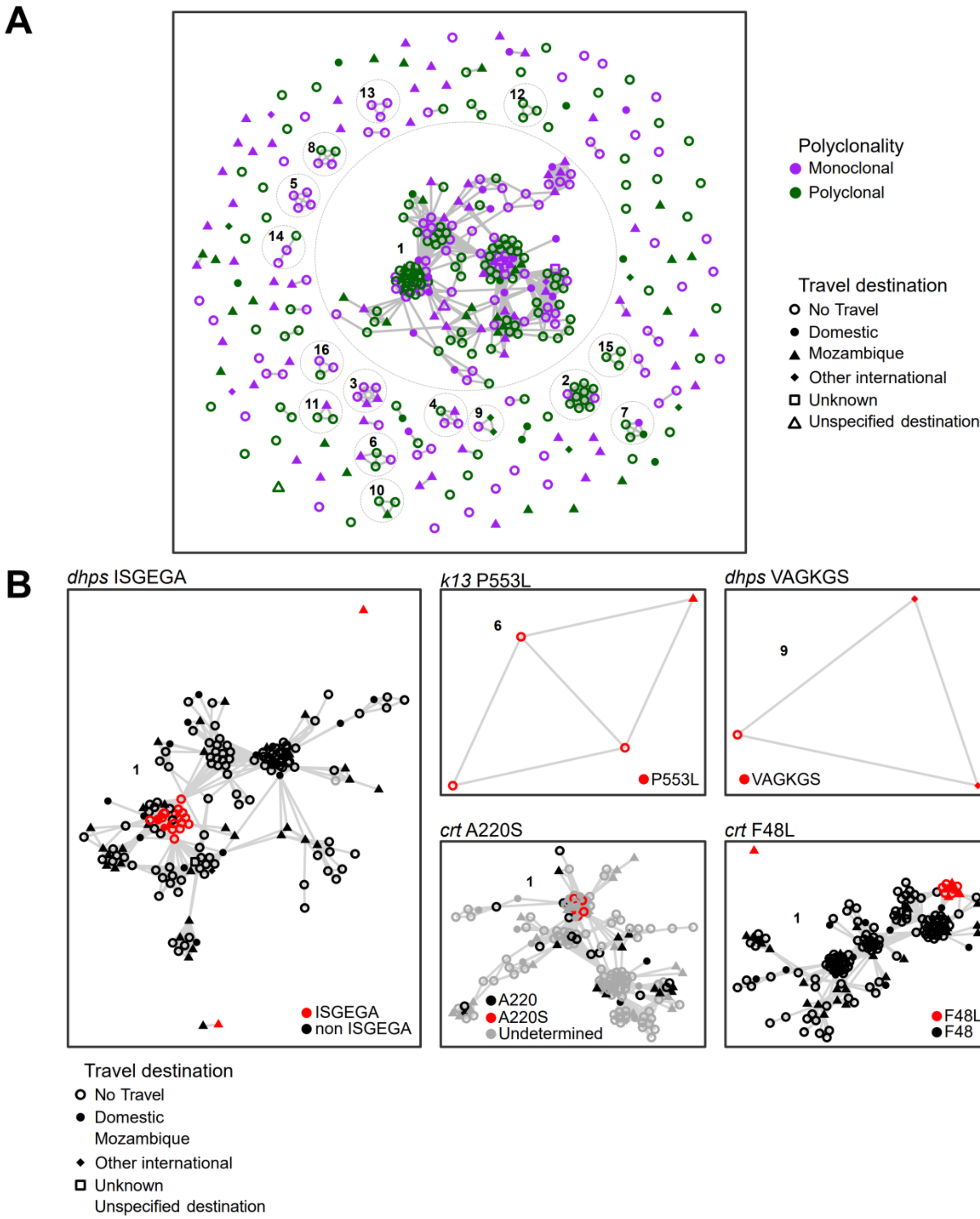

Figure S4: Clustering of samples with a relatedness threshold of 0.5. A. Clusters of genetically related samples, defined as sample pairs with a relatedness value of $\hat{r}$>0.5, at the 0.05 significance level. Only clusters composed of 3 or more samples are labeled and highlighted with a dashed outline. **B.** All clusters and isolated samples containing a genotype of interest are shown indicating the genotype for each sample in those clusters. Cluster labels are the same as panel A.

Table S1. Comparison of characteristics and origin of recruited RDT-positive participants and those with successfully sequenced samples

	RDT-positive	Successfully sequenced*	*P*-value**
N	572	379	
**Sex**			0.06
Female	156 (27%)	82 (22%)	
Male	415 (73%)	297 (78%)	
**Age**			
<5	16 (2.8%)	11 (2.9%)	0.97
5-14	85 (15%)	50 (13%)	
15-24	190 (34%)	128 (34%)	
25-39	177 (31%)	124 (33%)	
40-59	68 (12%)	47 (13%)	
>59	28 (5.0%)	16 (4.3%)	
**Occupation**			
Minor	24 (4.2%)	15 (4.0%)	0.68
Student	130 (23%)	82 (22%)	
Agricultural	243 (43%)	175 (46%)	
Other	55 (9.6%)	15 (4.0%)	
Unemployed	118 (21%)	66 (17%)	
**Nationality**			
Eswatini	478 (84%)	303 (80%)	0.18
Mozambique	94 (16%)	76 (20%)	
**Travel**			
No Travel	385 (67%)	245 (65%)	0.82
Domestic	46 (8.1%)	28 (7.4%)	
Mozambique	127 (22%)	95 (25%)	
Other Country	11 (1.9%)	9 (2.4%)	
Multiple	2 (0.4%)	2 (0.5%)	
**Detection method**			0.82
Passive	538 (94%)	353 (93%)	
Active	2 (0.3%)	2 (0.5%)	
Reactive	32 (5.6%)	24 (6.3%)	
**Region**			0.98
Hhohho	292 (51%)	196 (52%)	
Lubombo	209 (37%)	134 (35%)	
Manzini	62 (11%)	42 (11%)	
Shiselweni	9 (1.6%)	7 (1.8%)	

*Samples with a valid genotype in all 59 assessed *kelch13* SNPs.
**chi-squared test.

Table S2. Comparison of characteristics and origin of recruited participants who traveled to Mozambique to those who did not travel or traveled to other countries. This table only includes samples with valid genotypes in all 59 assessed *kelch13* SNPs

	No travel to Mozambique	Travel to Mozambique	*P*-value*
N	293	98	
**Sex**			0.005
Female	75 (26%)	11 (11%)	
Male	218 (74%)	87 (89%)	
**Age**			0.06
<5	10 (3.5%)	2 (2.0%)	
5-14	45 (15%)	5 (5.1%)	
15-24	92 (32%)	42 (43%)	
25-39	97 (34%)	29 (30%)	
40-59	33 (11%)	15 (15%)	
>59	11 (3.8%)	5 (5.1%)	
Unknown	3	0	
**Occupation**			<0.001
Minor	13 (4.5%)	3 (3.1%)	
Student	81 (28%)	3 (3.1%)	
Agricultural	113 (39%)	66 (67%)	
Other	19 (6.6%)	22 (22%)	
Unemployed	64 (22%)	4 (4.1%)	
Unknown	1	0	
**Detection method**			0.71
Passive	271 (93%)	92 (94%)	
Active	2 (0.7%)	0 (0%)	
Reactive	18 (6.2%)	6 (6.1%)	
**Region**			0.002
Hhohho	149 (51%)	49 (50%)	
Lubombo	113 (39%)	27 (28%)	
Manzini	23 (7.9%)	21 (21%)	
Shiselweni	6 (2.1%)	1 (1.0%)	

*chi-squared test.

Table S3. Logistic regression of infection polyclonality and case characteristics

	Univariate logistic regression			Multivariate logistic regression		
	n/N	**OR (95% CI)**	***P*-value**	***n/N* (%)**	**OR (95% CI)**	***P*-value**
**Age**						
<5	7/14 (50%)	Ref.		7/14 (50%)	Ref.	
5-14	37/59 (62.7%)	1.68 (0.51-5.55)	0.4	37/59 (62.7%)	1.78 (0.24-17.1)	0.6
15-24	98/154 (63.6%)	1.75 (0.57-5.36)	0.3	98/153 (62.7%)	3.13 (0.35-35.0)	0.3
25-39	81/149 (54.4%)	1.19 (0.39-3.64)	0.8	77/144 (53.5%)	2.53 (0.27-29.7)	0.4
40-59	29/52 (55.8%)	1.26 (0.38-4.19)	0.7	28/51 (54.9%)	2.93 (0.29-36.0)	0.4
60+	10/20 (50%)	1.00 (0.25-3.98)	>0.9	10/20 (50.0%)	2.99 (0.26-40.8)	0.4
**Sex**						
Female	66/106 (62.3%)	Ref.		61/101 (60.4%)	Ref.	
Male	201/350 (57.4%)	0.82 (0.52-1.27)	0.4	196/340 (57.6%)	0.96 (0.56-1.66)	0.9
**Occupation**						
Minor	11/20 (55%)	Ref.		11/20 (55%)	Ref.	
Student	61/95 (64.2%)	1.47 (0.54-3.90)	0.4	60/92 (65.2%)	0.81 (0.10-4.87)	0.8
Agricultural	121/209 (57.9%)	1.13 (0.44-2.83)	0.8	119/206 (57.8%)	0.35 (0.04-2.45)	0.3
Other	26/45 (57.8%)	1.12 (0.38-3.24)	0.9	25/43 (78.1%)	0.30 (0.03-2.34)	0.3
Unemployed	45/83 (54.2%)	0.97 (0.36-2.59)	>0.9	42/80 (52.5%)	0.31 (0.03-2.29)	0.3
**Detection method**						
Passive	247/427 (57.8%)	Ref.		225/384 (58.6%)	Ref.	
Proactive and reactive	16/25 (64.0%)	1.30 (0.57-3.12)	0.5	18/27 (66.7%)	1.01 (0.42-2.55)	0.8
**Travel History**						
No Travel	162/300 (54.0%)	Ref.		154/289 (53.3%)	Ref.	
Domestic	22/36 (61.1%)	1.34 (0.67-2.77)	0.4	22/36 (61.1%)	1.19 (0.55-2.67)	0.7
Mozambique	78/108 (72.2%)	2.21 (1.38-3.61)	0.001	78/107 (72.9%)	2.51 (1.45-4.45)	0.001
Other	3/9 (33.3%)	0.43 (0.09-1.65)	0.2	3/9 (33.3%)	0.48 (0.09-2.20)	0.4
**Parasitemia**						
log10(parasite density)	N=456	0.64 (0.54-0.75)	<0.001	N=441	0.72 (0.59-0.86)	<0.001
**Depth of coverage**						
log10(Reads)	N=456	0.42 (0.29-0.60)	<0.001	N=441	0.58 (0.39-0.87)	0.009

Table S4. Proportion of samples carrying a *dhps* or *dhfr* haplotype.
n: number of samples with the haplotype. *N*: total number of samples with a valid haplotype. CI: confidence interval.

n/*N*<br>%<br>[95% CI]	Overall	Hhohho	Lubombo	Manzini	Shiselweni
dhps					
ISAKAA[a]	10/429<br>2.3%<br>[1.1-4.2%]	5/227<br>2.2%<br>[0.7-5.1%]	4/149<br>2.7%<br>[0.7-6.7%]	0/47<br>0%	1/6<br>16.7%<br>[0.4-64.1%]
ISGKAA	8/429<br>1.9%<br>[0.8-3.6%]	2/227<br>0.9%<br>[0.1-3.1%]	3/149<br>2.0%<br>[0.4-5.8%]	3/47<br>6.4%<br>[1.3-17.5%]	0/6<br>0%
ISAEAA	3/429<br>0.7%<br>[0.1-2%]	0/227<br>0%	2/149<br>1.3%<br>[0.2-4.8%]	1/47<br>2.1%<br>[0.1-11.3%]	0/6<br>0%
ISGEAA[b]	405/429<br>94.4%<br>[91.8-96.4%]	219/227<br>96.5%<br>[93.2-98.5%]	140/149<br>94.0%<br>[88.8-97.2%]	42/47<br>89.4%<br>[76.9-96.5%]	4/6<br>66.7%<br>[22.3-95.7%]
ISGEGA[c]	29/429<br>6.8%<br>[4.6-9.6%]	10/227<br>4.4%<br>[2.1-8.0%]	13/149<br>8.7%<br>[4.7-14.5%]	6/47<br>12.8%<br>[4.8-25.7%]	0/6<br>0%
VAGKGS	3/429<br>0.7%<br>[0.1-2%]	1/227<br>0.4%<br>[0.0-2.4%]	0/141<br>0%	1/47<br>2.1%<br>[0.1%]	1/6<br>16.7%<br>[0.4-64.1%]
Undetermined[d]	22	6	13	2	1
dhfr					
NCSI	1/453<br>0.2%<br>[0-1.2%]	1/235<br>0.4%<br>[0-2.3%]	0/162<br>0%	0/48<br>0%	0/7<br>0%
NRNI	20/453<br>4.4%<br>[2.7-6.7%]	5/235<br>2.1%<br>[0.7-4.9%]	13/162<br>8.0%<br>[4.3-13.3%]	2/49<br>4.2%<br>[0.5-14.0%]	0/7<br>0%
IRNI[e]	448/453<br>98.9%<br>[97.4-99.6%]	232/235<br>98.7%<br>[96.3-99.7%]	160/162<br>98.8%<br>[95.6-99.9%]	49/49<br>100%<br>[92.7-100%]	7/7<br>100%<br>[59-100%]
Undetermined[d]	1	1	0	0	0

[a]: wild-type haplotype. [b]: *dhps* double mutant. [c]: *dhps* triple mutant. [d]: samples with mixed genotypes in two or more targets. [e]: *dhfr* triple mutant.

Table S5. Logistic regression of *dhps/dhfr* quintuple mutant carriage and case characteristics

	Univariate logistic regression			Multivariate logistic regression		
	***n*/*N* (%)**	**OR (95% CI)**	***P*-value**	***n*/*N* (%)**	**OR (95% CI)**	***P*-value**
**Age**						
<15	64/72 (88.9%)	Ref.		64/72 (88.9%)	Ref.	
15-24	142/155 (91.6%)	1.37 (0.52-3.40)	0.5	141/154 (91.6%)	1.44 (0.40-5.38)	0.6
25-39	122/143 (85.3%)	0.73 (0.29-1.67)	0.5	117/138 (84.8%)	0.52 (0.11-2.35)	0.4
40+	64/73 (87.7%)	0.89 (0.32-2.47)	0.8	63/72 (87.5%)	1.29 (0.25-6.89)	0.8
**Sex**						
Female	90/106 (84.9%)	Ref.		85/101 (84.2%)	Ref.	
Male	310/345 (89.9%)	1.57 (0.81-2.93)	0.2	300/335 (89.6%)	1.1 (0.50-2.35)	0.8
**Occupation**						
Minor	19/20 (95%)	Ref.		19/20 (95%)	Ref.	
Student	81/93 (87.1%)	0.36 (0.02-1.98)	0.3	78/90 (86.7%)	0.33 (0.02-2.12)	0.3
Agricultural	192/207 (92.8%)	0.67 (0.04-3.62)	0.7	189/204 (92.6%)	1.03 (0.04-10.1)	>0.9
Other	39/45 (86.7%)	0.34 (0.02-2.20)	0.3	37/43 (86%)	0.58 (0.02-6.89)	0.7
Unemployed	65/82 (79.3%)	0.2 (0.01-1.08)	0.13	62/79 (78.5%)	0.18 (0.01-1.75)	0.2
**Detection method**						
Passive	374/422 (88.6%)	Ref.		363/411 (88.3%)	Ref.	
Proactive and reactive	22/25 (88%)	0.94 (0.31-4.08)	>0.9	22/25 (88%)	1.2 (0.36-5.78)	0.8
**Travel History**						
No Travel	264/294 (89.8%)	Ref.		253/283 (89.4%)	Ref.	
Domestic	34/37 (91.9%)	1.29 (0.43-5.57)	0.7	34/37 (91.9%)	1.65 (0.50-7.55)	0.5
Mozambique	96/108 (88.9%)	0.91 (0.46-1.91)	0.8	95/107 (88.8%)	0.51 (0.21-1.23)	0.13
Other	3/9 (33.3%)	0.06 (0.01-0.23)	<0.001	3/9 (33.3%)	0.05 (0.01-0.23)	<0.001
**Parasitemia**						
log10(Reads)	*N* = 449	1.03 (0.82-1.28)	0.8	*N* = 434	1.07 (0.83-1.36)	0.6

Table S6. Logistic regression of *dhps*/*dhfr* sextuple mutant carriage and case characteristics

	Univariate logistic regression			Multivariate logistic regression		
	***n*/*N* (%)**	**OR (95% CI)**	***P*-value**	***n*/*N* (%)**	**OR (95% CI)**	***P*-value**
**Age**						
<15	4/72 (5.6%)	Ref.		4/71 (5.6%)	Ref.	
15-24	11/155 (7.1%)	1.3 (0.43-4.82)	0.7	11/151 (7.3%)	1.64 (0.52-6.30)	0.4
25-39	11/143 (7.7%)	1.42 (0.46-5.26)	0.6	11/135 (8.1%)	1.7 (0.54-6.53)	0.4
40+	2/73 (2.7%)	0.48 (0.06-2.54)	0.4	2/70 (2.9%)	0.63 (0.08-3.42)	0.6
**Sex**						
Female	9/106 (8.5%)	Ref.		8/99 (8.1%)	Ref.	
Male	20/345 (5.8%)	0.66 (0.30-1.58)	0.3	20/328 (6.1%)	0.79 (0.33-2.05)	0.6
**Travel History**						
No Travel	24/294 (8.2%)	Ref.		23/283 (8.1%)	Ref.	
Domestic	2/37 (5.4%)	0.64 (0.10-2.30)	0.6	2/37 (5.4%)	0.59 (0.09-2.19)	0.5
Mozambique	3/108 (2.8%)	0.32 (0.08-0.94)	0.069	3/107 (2.8%)	0.29 (0.07-0.89)	0.053
**Parasitemia**						
log10(Reads)	*N* = 449	0.81 (0.62-1.07)	0.13	*N* = 429	0.78 (0.59-1.06)	0.1

Table S7. Logistic regression of *mdr1* F184Y and case characteristics

	Univariate logistic regression			Multivariate logistic regression		
	***n*/*N* (%)**	**OR (95% CI)**	***P*-value**	***n*/*N* (%)**	**OR (95% CI)**	***P*-value**
**Age**						
<5	10/13 (76.9%)	Ref.		10/13 (76.9%)	Ref.	
5-14	35/58 (60.3%)	0.46 (0.09-1.68)	0.3	35/58 (60.3%)	0.14 (0.01-1.09)	0.073
15-24	80/149 (53.7%)	0.35 (0.08-1.19)	0.12	80/148 (54.1%)	0.1 (0.01-0.94)	0.052
25-39	83/140 (59.3%)	0.44 (0.09-1.50)	0.2	78/135 (57.8%)	0.14 (0.01-1.34)	0.1
40-59	30/53 (56.6%)	0.39 (0.08-1.45)	0.2	29/52 (55.8%)	0.11 (0.01-1.15)	0.075
60+	13/20 (65%)	0.56 (0.10-2.58)	0.5	13/20 (65%)	0.18 (0.01-2.15)	0.2
**Sex**						
Female	59/100 (59%)	Ref.		56/96 (58.3%)	Ref.	
Male	195/340 (57.4%)	0.93 (0.59-1.47)	0.8	189/330 (57.3%)	0.99 (0.58-1.67)	>0.9
**Occupation**						
Minor	12/19 (63.2%)	Ref.		12/19 (63.2%)	Ref.	
Student	55/93 (59.1%)	0.84 (0.29-2.30)	0.7	55/91 (60.4%)	4.08 (0.71-32.0)	0.13
Agricultural	114/202 (56.4%)	0.76 (0.27-1.96)	0.6	111/199 (55.8%)	2.84 (0.43-24.7)	0.3
Other	27/42 (64.3%)	1.05 (0.33-3.21)	>0.9	25/40 (62.5%)	3.54 (0.48-33.6)	0.2
Unemployed	44/80 (55%)	0.71 (0.24-1.96)	0.5	42/77 (54.5%)	2.96 (0.43-26.5)	0.3
**Detection method**						
Passive	234/411 (56.9%)	Ref.		228/401 (56.9%)	Ref.	
Proactive and reactive	17/25 (68%)	1.61 (0.70-4.02)	0.3	17/25 (68%)	1.75 (0.74-4.50)	0.2
**Travel History**						
No Travel	158/287 (55.1%)	Ref.		152/277 (54.9%)	Ref.	
Domestic	22/34 (64.7%)	1.5 (0.72-3.23)	0.3	22/34 (64.7%)	1.55 (0.72-3.45)	0.3
Mozambique	68/107 (63.6%)	1.42 (0.90-2.26)	0.13	68/106 (64.2%)	1.68 (1.00-2.87)	0.054
Other	3/9 (33.3%)	0.41 (0.08-1.58)	0.2	3/9 (33.3%)	0.33 (0.07-1.35)	0.14
**Parasitemia**						
log10(parasite density)	*N* = 430	1 (0.87-1.16)	>0.9	*N* = 426	1.06 (0.91-1.24)	0.4

Table S8. Logistic regression of *mdr2* I492V and case characteristics

	Univariate logistic regression			Multivariate logistic regression		
	***n*/*N* (%)**	**OR (95% CI)**	***P*-value**	***n*/*N* (%)**	**OR (95% CI)**	***P*-value**
**Age**						
<5	2/11 (18.2%)	Ref.		2/11 (18.2%)	Ref.	
5-14	12/51 (23.5%)	1.38 (0.30-9.90)	0.7	12/51 (23.5%)	1.4 (0.05-21.3)	0.8
15-24	27/138 (19.6%)	1.09 (0.26-7.45)	>0.9	27/138 (19.6%)	1.08 (0.04-19.5)	>0.9
25-39	39/127 (30.7%)	1.99 (0.49-13.5)	0.4	39/122 (32%)	2.16 (0.07-41.2)	0.6
40-59	15/45 (33.3%)	2.25 (0.50-16.0)	0.3	15/44 (34.1%)	2.64 (0.08-52.9)	0.5
60+	5/15 (33.3%)	2.25 (0.38-18.6)	0.4	5/15 (33.3%)	2.79 (0.08-65.8)	0.5
**Sex**						
Female	18/83 (21.7%)	Ref.		18/81 (22.2%)	Ref.	
Male	82/309 (26.5%)	1.3 (0.74-2.38)	0.4	82/300 (27.3%)	1.21 (0.63-2.39)	0.6
**Occupation**						
Minor	3/15 (20%)	Ref.		3/15 (20%)	Ref.	
Student	18/85 (21.2%)	1.07 (0.30-5.08)	>0.9	18/83 (21.7%)	0.91 (0.10-19.6)	>0.9
Agricultural	53/184 (28.8%)	1.62 (0.49-7.31)	0.5	53/181 (29.3%)	0.88 (0.08-20.6)	>0.9
Other	11/39 (28.2%)	1.57 (0.40-7.86)	0.5	11/37 (29.7%)	0.73 (0.06-18.5)	0.8
Unemployed	15/66 (22.7%)	1.18 (0.32-5.65)	0.8	15/65 (23.1%)	0.67 (0.06-16.6)	0.8
**Detection method**						
Passive	93/365 (25.5%)	Ref.		93/358 (26%)	Ref.	
Proactive and reactive	7/23 (30.4%)	1.28 (0.48-3.10)	0.6	7/23 (30.4%)	1.21 (0.44-3.02)	0.7
**Travel History**						
No Travel	58/253 (22.9%)	Ref.		58/245 (23.7%)	Ref.	
Domestic	7/29 (24.1%)	1.07 (0.41-2.52)	0.9	7/29 (24.1%)	1.05 (0.38-2.63)	>0.9
Mozambique	31/99 (31.3%)	1.53 (0.91-2.56)	0.1	31/99 (31.3%)	1.37 (0.75-2.49)	0.3
Other	4/8 (50%)	3.36 (0.77-14.6)	0.093	4/8 (50%)	3.55 (0.78-16.4)	0.092
**Parasitemia**						
log10(parasite density)	*N* = 392	0.9 (0.76-1.07)	0.2	*N* = 381	0.87 (0.72-1.05)	0.13
